# Inflammatory biomarkers and immune dysregulation in gestational diabetes mellitus: emerging mechanisms and therapeutic prospects

**DOI:** 10.3389/fimmu.2026.1953251

**Published:** 2026-10-30

**Authors:** Qian Zhang, Yang Shu, Yanan Gao

**Affiliations:** 1 Department of Obstetrics, Obstetrics and Gynaecology Center, The First Hospital of Jilin University, Changchun, Jilin, China; 2 Department of Breast Surgery, General Surgery Center, The First Hospital of Jilin University, Changchun, Jilin, China

**Keywords:** C-reactive protein, cytokines, gestational diabetes mellitus, inflammatory biomarker, insulin resistance, white blood cell count

## Abstract

Gestational diabetes mellitus (GDM) is a common pregnancy-related metabolic disorder that increases the risk of adverse maternal and neonatal outcomes and predisposes both mothers and offspring to long-term metabolic disease. Beyond pregnancy-induced insulin resistance, accumulating evidence indicates that GDM is shaped by chronic low-grade inflammation and immune dysregulation at systemic, adipose, and placental levels. Pro-inflammatory cytokines, including tumor necrosis factor-α, interleukin-6, and interleukin-1β, disrupt insulin receptor signaling through NF-κB, JAK/STAT–SOCS3, mTOR, and inflammasome-related pathways, thereby amplifying insulin resistance and β-cell stress. Acute-phase proteins such as high-sensitivity C-reactive protein, pentraxin 3, serum amyloid A, and procalcitonin reflect distinct layers of inflammatory activation and may provide complementary value for early risk stratification, although their diagnostic performance is influenced by gestational timing, maternal adiposity, and population heterogeneity. Alterations in leukocyte profiles, neutrophil-to-lymphocyte ratio, monocyte/macrophage activation, Treg dysfunction, Th1/Th17 skewing, oxidative stress, and neutrophil extracellular trap formation further reveal the immune imbalance underlying GDM progression. This review synthesizes current evidence on inflammatory biomarkers and immune mechanisms in GDM, emphasizing their predictive potential, mechanistic relevance, and translational implications for biomarker-guided diagnosis, individualized risk assessment, and future anti-inflammatory interventions.

## 1 Introduction

Gestational diabetes mellitus (GDM), characterized by glucose intolerance first diagnosed during pregnancy, constitutes one of the most prevalent metabolic complications of gestation worldwide, with an estimated global prevalence of 14% (1–3). This condition is robustly associated with adverse maternal-neonatal outcomes, including preeclampsia, preterm delivery, macrosomia, and neonatal hypoglycemia (4, 5). Critically, GDM imparts enduring health consequences: affected women exhibit a nearly tenfold elevated risk of subsequent type 2 diabetes (6), while their offspring demonstrate increased susceptibility to obesity and metabolic syndrome in adulthood (7). The high recurrence rate in subsequent pregnancies (up to 56%) further amplifies the public health imperative for refined risk prediction and targeted intervention (8). Converging evidence implicates chronic, low-grade systemic inflammation as a pivotal pathophysiological mechanism in GDM (9, 10). Pro-inflammatory mediators are thought to exacerbate insulin resistance through multiple molecular pathways, thereby contributing to the onset and progression of GDM. Inflammatory biomarkers—encompassing pro-inflammatory cytokines, acute-phase inflammatory proteins, and hematological indices reflective of inflammatory status—have consequently emerged as potential indicators linked to GDM risk and pathogenesis (11, 12). This review outlines current evidence on the association between inflammatory biomarkers and GDM, with the aim of providing an integrated theoretical foundation to inform future strategies for the diagnosis, management, and prevention of this condition.

## 2 Pro-inflammatory cytokines

### 2.1 Tumor necrosis factor-α

In a first-trimester cohort of 205 singleton pregnancies, including 20 women who subsequently developed GDM, multimarker models incorporating inflammatory, metabolic, and placental factors demonstrated high discriminatory performance. Among women with obesity, the combination of BMI, insulin, soluble CD163, and TNF-α achieved an AUC of 0.95, with an 89% detection rate at a 10% false-positive rate. In women without obesity, a model combining insulin, soluble CD163, TNF-α, placental protein 13, and pregnancy-associated plasma protein-A achieved an AUC of 0.94, with an 83% detection rate at the same false-positive rate (13). Although these findings support the potential value of integrating TNF-α into multimarker prediction models, their interpretation is limited by the small number of GDM cases and the absence of external validation. Beyond its diagnostic utility, the involvement of TNF-α in GDM pathogenesis is supported by epigenetic studies, which have identified altered methylation patterns in the TNF-α gene in GDM (14). Furthermore, genetic association studies indicate that the TNF-α rs1800629 A allele increases GDM susceptibility in Asian populations (15). Mechanistically, experimental studies suggest that hydrogen sulfide (H_2_S) can suppress insulin secretion and β-cell activity; notably, decreased H_2_S levels coincide with elevated TNF-α under hyperglycemic conditions, pointing to a potential interaction within the inflammatory cascade of GDM (16). Elevated TNF-α in early pregnancy has also been correlated with increased placental secretion of granulocyte-macrophage colony-stimulating factor, chemokine ligand 5, and IL-10, implying the activation of a compensatory anti-inflammatory response (17). Collectively, these findings underscore a multifaceted role for TNF-α in GDM pathophysiology that merits further investigation.

### 2.2 Interleukins

Elevated circulating levels of IL-6 are consistently reported in individuals with GDM, reinforcing its involvement in the low-grade inflammatory state characteristic of the disorder (18, 19). Genetic susceptibility also plays a role, as the rs1800796 G allele in the IL-6 gene is significantly associated with increased GDM risk (15, 20). Mechanistic insights suggest that IL-6 activity may be modulated by hydrogen sulfide (H_2_S), given the observed inverse correlation between their concentrations, hinting at a regulatory interaction within GDM-related inflammation (16, 21). Furthermore, hyperglycemia has been shown to impair trophoblast function through the miR-137/PRKAA1/IL-6 axis, thereby contributing to placental dysfunction in GDM (22, 23). In contrast to IL-6, polymorphisms in the IL-1β gene do not appear to confer overall susceptibility to GDM. Nevertheless, among patients who require insulin therapy, the rs16944 AA genotype is associated with both a higher frequency and a greater magnitude of insulin requirement (24). In murine GDM model, anti-IL-1β antibody treatment improved maternal glucose tolerance without adverse fetal effects (25). *In vitro*, work by Negi et al. (26) demonstrated that allopurinol attenuates the high-glucose-induced pro-inflammatory and anti-angiogenic responses in trophoblasts by suppressing IL-1β and reactive oxygen species production. This suggests that allopurinol could represent a candidate therapeutic for preventing placental dysfunction and associated complications, such as preeclampsia, in GDM. These findings indicate that IL-1β contributes to impaired glucose homeostasis in GDM, and its targeted inhibition may alleviate insulin resistance and inflammation. This provides a conceptual foundation for the development of novel adjunctive therapies aimed at improving pregnancy outcomes in affected individuals (**Supplementary Table S1**).

### 2.3 Pro-inflammatory factors mediate insulin resistance

IL-1β, IL-6, and TNF-α directly impair insulin signaling and promote insulin resistance (IR), particularly in obesity, where chronic low-grade inflammation exacerbates cytokine dysregulation (19, 27). Higher concentrations of TNF-α and IL-6 have been observed in umbilical cord blood of obese women with GDM compared to their normal-BMI counterparts (28). Moreover, maternal gestational weight gain, reflected by an increase in BMI, is positively correlated with rising levels of circulating inflammatory mediators (29, 30). These cytokines primarily promote IR through the NF-κB and JAK/STAT pathways (31, 32). TNF-α, IL-6, and IL-1β induce IκB phosphorylation and degradation, enabling NF-κB nuclear translocation and transcription of inflammatory genes that disrupt insulin action (33, 34). IL-6 additionally activates the JAK/STAT3 pathway, leading to STAT3 phosphorylation and induction of suppressor of cytokine signaling 3 (SOCS3). SOCS3 inhibits insulin receptor and IRS tyrosine phosphorylation and promotes their ubiquitin-mediated degradation (35, 36). The STAT3–SOCS3 axis also interacts with mammalian target of rapamycin (mTOR) signaling, further reinforcing IR and metabolic dysregulation in GDM (37).

Inflammatory dysregulation in GDM is orchestrated by a broader network of innate immune sensing and metabolic signaling pathways. Notably, activation of the NLRP3 inflammasome has been implicated as a central driver linking metabolic stress to sterile inflammation (38, 39). Hyperglycemia and lipid accumulation induce mitochondrial dysfunction and ROS generation, triggering NLRP3 activation in macrophages and placental trophoblasts (40–42). Subsequent caspase-1 activation promotes maturation of IL-1β and IL-18, amplifying inflammation and impairing systemic and placental insulin signaling (43, 44). In parallel, aberrant activation of the cGAS–STING pathway has recently emerged as a critical mediator of metabolic inflammation (45). Cellular stress and mitochondrial DNA leakage in GDM can activate cGAS and induce STING-dependent type I interferon responses (46, 47). This pathway enhances pro-inflammatory cytokine production and may interfere with insulin receptor signaling through interferon-mediated suppressive transcriptional programs, thereby linking metabolic stress, innate immune activation, and chronic inflammation in GDM (48).

## 3 Acute inflammation-related proteins

### 3.1 C-reactive protein and high-sensitivity C-reactive protein

Chemically, high-sensitivity CRP (hs-CRP) is identical to conventional CRP but is measured with enhanced analytical sensitivity, rendering it a valuable biomarker for detecting chronic, low-grade inflammatory states (49, 50). CRP is primarily synthesized by hepatocytes in response to inflammatory cytokines, particularly IL-6, with additional regulation by interleukin-1β and tumor necrosis factor-α (19, 51, 52). Therefore, elevated CRP or hs-CRP in GDM should be interpreted not as an isolated pathogenic factor, but as an integrated downstream readout of systemic inflammatory activation. Accumulating evidence suggests that both CRP and hs-CRP may serve as potential predictive markers for GDM. Notably, hs-CRP represents a cost-effective and accessible inflammatory marker for GDM risk assessment (53, 54). Longitudinal studies indicate that hs-CRP levels in individuals who subsequently develop GDM are significantly elevated beginning in the second trimester compared with normoglycemic pregnancies, with further increases during the third trimester (55). Consequently, elevated second-trimester hs-CRP is regarded as an independent risk factor for GDM and may also function as a predictor for impaired glucose tolerance 6 to 12 weeks postpartum (56, 57). However, the clinical interpretation of hs-CRP requires caution (53, 58). Unlike cytokines such as TNF-α, IL-6, and IL-1β, which can directly interfere with insulin receptor signaling, CRP is more accurately positioned as a surrogate marker of upstream inflammatory pathways rather than a direct mechanistic driver of insulin resistance (59, 60). Its association with GDM may reflect activation of the IL-6–hepatic acute-phase response, adipose tissue inflammation, endothelial dysfunction, and oxidative stress.

### 3.2 Pentraxin 3

Pentraxin 3 (PTX3), an acute-phase protein produced by macrophages, neutrophils, endothelial cells, and adipocytes in response to inflammatory stimuli, shares structural homology with CRP and functions in the regulation of innate immunity, inflammation, and tissue remodeling (61, 62). Unlike CRP, which mainly reflects the hepatic acute-phase response, PTX3 is generated locally at sites of inflammation and tissue remodeling. In GDM, PTX3 may provide a more tissue-proximal readout of placental, vascular, and adipose inflammatory activation rather than merely indicating systemic inflammation (63, 64). Clinical evidence regarding the predictive value of PTX3 remains heterogeneous. In a prospective cohort of 824 pregnant women, serum PTX3 was measured at 10–20 weeks of gestation, with a median sampling time of 14.86 weeks. A PTX3 threshold of 1.21 ng/mL yielded high sensitivity (94.6%) but low specificity (20.2%) for subsequent GDM. Importantly, the reported AUC of 0.743 was achieved by a combined model incorporating maternal characteristics, PTX3, HbA1c, and fasting insulin rather than by PTX3 alone. The AUC increased from 0.602 for maternal characteristics to 0.657 after adding PTX3 and to 0.743 after including the biochemical markers (65, 66). By contrast, another longitudinal study found no significant difference in PTX3 during early pregnancy but observed higher concentrations in women with GDM during the second and third trimesters (55, 67). Other cohorts have reported lower early-pregnancy PTX3 concentrations among women who subsequently developed GDM (68) (**Supplementary Table S2**). Thus, PTX3 represents a promising inflammatory biomarker in GDM.

### 3.3 Serum amyloid A and procalcitonin

Serum amyloid A (SAA), an acute-phase inflammatory apolipoprotein predominantly synthesized by hepatocytes, has been increasingly implicated in metabolic dysregulation (69, 70). In addition to its hepatic origin, SAA can be secreted by adipocytes in response to hyperglycemic conditions *in vitro*, linking it mechanistically to obesity and insulin resistance (IR) (70, 71). Serum amyloid A (SAA), particularly SAA1, has been shown to engage TLR2 and/or TLR4 in a context-dependent manner (72, 73), thereby activating downstream inflammatory signaling pathways (74–76). SAA may contribute to NLRP3 inflammasome activation, enhancing IL-1β maturation and amplifying inflammatory cascades (77, 78). These processes can exacerbate oxidative stress and impair insulin signaling pathways, suggesting that SAA may function not only as a biomarker but also as a potential mediator of metabolic dysfunction in GDM (70). Clinical investigations have demonstrated that circulating SAA concentrations are elevated in patients with GDM during the 24–32 weeks of gestation compared to normoglycemic controls (70). Moreover, SAA levels exhibit significant correlations with clinical and metabolic parameters such as maternal age, body mass index (BMI), mean arterial pressure, glucose tolerance indices, and carotid intima-media thickness. These associations suggest that SAA may serve not only as a biomarker of metabolic derangement but also as a predictive indicator of subclinical atherosclerosis and heightened cardiovascular risk in GDM populations (79). The paucity of research specifically addressing the role of SAA in obese pregnancies with GDM and its dynamic relationship with glycemic control represents a critical gap warranting future investigation. PCT, another inflammatory biomarker, is well-established for its diagnostic accuracy in systemic infections due to its high sensitivity and specificity (80, 81). Interestingly, a retrospective study comparing late gestational PCT levels in GDM patients revealed significantly elevated PCT concentrations in the GDM cohort (82).

## 4 Immune cells in gestational diabetes mellitus

White blood cells (WBCs), including neutrophils, monocytes, and lymphocytes, constitute major cellular components of innate and adaptive immunity (83, 84). In GDM, increased WBC counts are commonly interpreted as a marker of systemic low-grade inflammation (85). In a case-control study conducted at 24–28 weeks of gestation, WBC count showed limited discrimination between women with and without GDM (AUC 0.643). In the same population, plateletcrit demonstrated better diagnostic performance, with an AUC of 0.766; at a threshold of 0.20%, its sensitivity and specificity were 62.7% and 78.2%, respectively (86–88). These findings suggest that routine hematological indices may complement, but cannot replace, established glucose-based diagnostic testing. Elevated WBC counts may indicate activation of innate immune pathways that contribute to insulin resistance. Activated neutrophils and monocytes can produce TNF-α, IL-6, IL-1β, ROS, and chemokines such as MCP-1/CCL2 (89). These mediators activate NF-κB, JNK, and inflammasome-related pathways, thereby impairing insulin receptor signaling and amplifying systemic inflammation (89). Notably, elevated WBC levels were associated with insulin requirement among patients with GDM, and WBC count has been positively correlated with insulin resistance (90, 91).

### 4.1 Neutrophils and lymphocytes

Neutrophils are key effector cells of the innate immune system and represent the dominant leukocyte subset contributing to early inflammatory responses. Epidemiological evidence consistently indicates that neutrophilia precedes clinical diagnosis and prediction: circulating neutrophil counts exceeding 5.0 × 10^9^/L in early gestation independently predict GDM onset (92, 93), while prospective cohort data reveal a 1.9-fold increased risk among women with elevated neutrophil levels prior to 14 weeks of gestation (94, 95). Neutrophil activation appears to play a more critical role in GDM pathophysiology. Pro-inflammatory cytokines such as TNF-α and IL-6 can prime neutrophils, leading to activation of NADPH oxidase and increased production of ROS. This oxidative burst facilitates neutrophil extracellular trap (NET) formation and subsequent elastase release, which interferes with insulin signaling by impairing insulin receptor substrate (IRS) function and downstream PI3K–AKT signaling (96, 97).

Lymphocytes, including T cells, B cells, and NK cells, play essential roles in adaptive immune regulation and maintenance of immune tolerance during pregnancy (98, 99). Epidemiological evidence regarding lymphocyte counts in GDM remains inconsistent. Some studies have reported elevated lymphocyte levels in early and mid-gestation among women who develop GDM (4), whereas others observed no differences between GDM and normoglycemic pregnancies (87, 88, 100). Central to this dysregulation is the impairment of Tregs, which normally maintain maternal–fetal immune tolerance through secretion of IL-10 and TGF-β and suppression of effector T-cell activation. In GDM, Treg frequency and suppressive capacity are diminished, resulting in unchecked secretion of IFN-γ and TNF-α by CD4^+^ and CD8^+^ effector subsets (101). Concomitantly, a pro-inflammatory skewing toward Th1 and Th17 phenotypes has been documented: Th17-derived IL-17 promotes neutrophil recruitment via CXCL1/2 induction, enhances endothelial activation, and exacerbates oxidative stress through NADPH oxidase upregulation (102, 103). This shift from a regulatory to a pro-inflammatory adaptive immune profile further amplifies the inflammatory milieu associated with GDM.

The neutrophil-to-lymphocyte ratio (NLR) serves as a robust and integrative marker of systemic inflammation and immune modulation. Elevated NLR in early gestation nearly doubles the likelihood of subsequent GDM development (94, 104), while second-trimester NLR elevation confers a 3.1-fold increased risk (105). A retrospective case-control study reported that NLR differentiated women with hyperglycemia first detected during pregnancy from normoglycemic controls with an AUC of 0.78. An NLR threshold above 4.394 was associated with greater clinical severity and adverse perinatal outcomes (106), underscoring its potential utility in risk stratification. The predictive superiority of NLR over isolated cell counts likely reflects its capacity to capture the dynamic equilibrium between pro-inflammatory (neutrophil-driven) and immunoregulatory (lymphocyte-mediated) forces. In GDM, an elevated NLR may signify not only heightened innate immune activation but also compromised adaptive immune tolerance—a dual perturbation that potentiates insulin resistance through synergistic cytokine release, oxidative stress, and direct interference with insulin receptor signaling (107). Consequently, NLR represents a clinically accessible, cost-effective biomarker that encapsulates the immuno-metabolic interface central to GDM pathogenesis (**Figure 1**).

**Figure 1:**
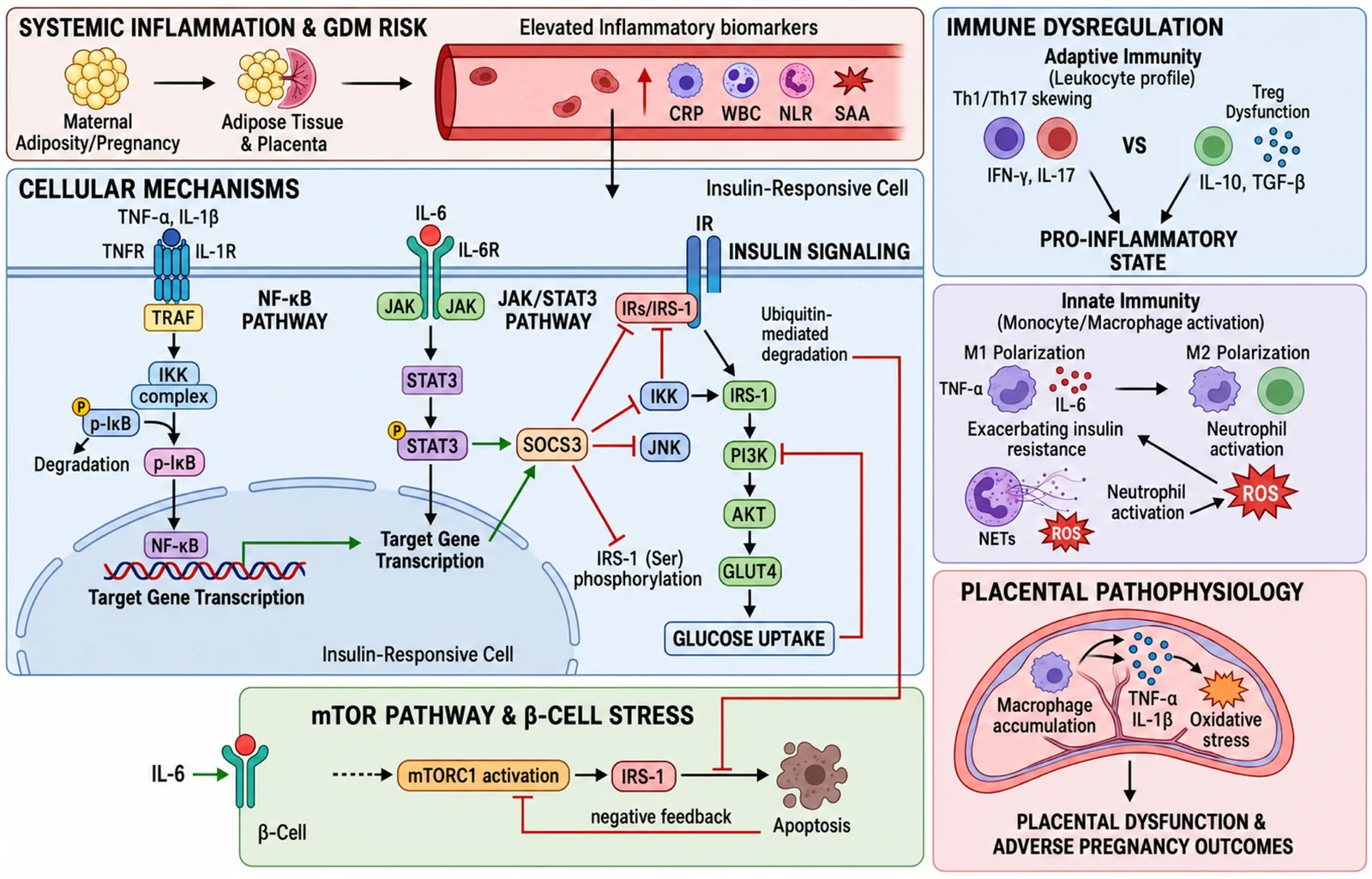
Inflammatory biomarkers and immune dysregulation in gestational diabetes mellitus.

### 4.2 Monocytes

Monocytes are key components of the innate immune system and serve as precursors to tissue macrophages and dendritic cells, playing a central role in inflammation, immune regulation, and metabolic homeostasis (108, 109). Circulating monocytes can be recruited to metabolic tissues and the placenta, where they differentiate into macrophages with either pro-inflammatory (M1-like) or anti-inflammatory (M2-like) phenotypes (21). In GDM, a shift toward pro-inflammatory M1 polarization has been proposed, leading to increased production of cytokines such as TNF-α, IL-6, and IL-1β, which contribute to insulin resistance (110). MCP-1 promotes monocyte recruitment via its receptor CCR2 and enhances inflammatory signaling within adipose tissue and the placenta. Activation of the MCP-1/CCR2 axis can trigger downstream pathways including extracellular signal-regulated kinase (ERK) and NF-κB signaling, leading to reduced tyrosine phosphorylation of the insulin receptor and impaired insulin signaling (111, 112). In addition, monocyte-derived cytokines can amplify local and systemic inflammation, reinforcing insulin resistance through JNK and inflammasome-related pathways (21, 97). In adipose tissue, infiltrating monocyte-derived macrophages contribute to chronic low-grade inflammation by forming crown-like structures around hypertrophic adipocytes and releasing pro-inflammatory mediators (113, 114). In the placenta, monocyte/macrophage accumulation may disrupt trophoblast function, promote endothelial dysfunction, and alter nutrient transport, thereby linking immune activation to adverse pregnancy outcomes (21, 115, 116). Reduction in monocyte counts during early gestation correlates with an increased risk of GDM. Specifically, monocyte levels in early pregnancy are associated with heightened susceptibility to GDM onset (117). By mid-gestation (24–28 weeks), monocyte counts tend to be elevated in GDM cases compared to non-GDM controls (106), although other investigations have failed to identify a statistically significant distinction between the two groups (87). These inconsistencies may reflect the temporal fluctuations in monocyte levels that naturally occur throughout pregnancy (118).

## 5 Conclusion

Gestational diabetes mellitus is increasingly recognized as a disorder underpinned by immune-metabolic dysregulation, wherein chronic low-grade inflammation serves as a critical driver of insulin resistance. Pro-inflammatory cytokines, including TNF-α, IL-6, and IL-1β, compromise insulin signaling through canonical NF-κB, JAK/STAT, and SOCS-dependent pathways, while acute-phase proteins (hs-CRP, PTX3) and hematological indices (NLR, WBC subsets) provide integrative readouts of systemic inflammatory burden. Nevertheless, heterogeneity in study designs, biomarker timing, and diagnostic criteria has yielded inconsistent associations, underscoring the need for standardized, longitudinal cohorts with harmonized analytical protocols. Therapeutically, targeting specific inflammatory axes—such as IL-1β neutralization or modulation of hydrogen sulfide signaling—holds promise for adjunctive intervention. By bridging mechanistic discovery with clinical translation, inflammatory biomarker research may ultimately enable precision risk stratification and personalized management strategies to mitigate the short- and long-term sequelae of GDM for both mother and offspring.
